# Vitamin D Deficiency as a Context-Dependent Modifier of Osteonecrosis of the Jaw

**DOI:** 10.3390/nu18111769

**Published:** 2026-05-30

**Authors:** Chien-Lin Lu, Ren-Yeong Huang, Cai-Mei Zheng, Kuo-Cheng Lu

**Affiliations:** 1 School of Medicine, College of Medicine, Fu Jen Catholic University, New Taipei City 24205, Taiwan; 096195@mail.fju.edu.tw; 2 Division of Nephrology, Department of Internal Medicine, Fu Jen Catholic University Hospital, Fu Jen Catholic University, New Taipei City 24352, Taiwan; 3 Department of Periodontology, School of Dentistry, Tri-Service General Hospital, National Defense Medical University, Taipei City 11490, Taiwan; 4 Division of Nephrology, Department of Internal Medicine, Shuang Ho Hospital, Taipei Medical University, New Taipei City 23561, Taiwan; 5 Division of Nephrology, Department of Internal Medicine, School of Medicine, College of Medicine, Taipei Medical University, New Taipei City 11031, Taiwan; 6 TMU Research Center of Urology and Kidney, Taipei Medical University, New Taipei City 11031, Taiwan; 7 Division of Nephrology, Department of Medicine, Taipei Tzu Chi Hospital, Buddhist Tzu Chi Medical Foundation, New Taipei City 23142, Taiwan

**Keywords:** angiogenesis, bone remodeling, immune regulation, osteonecrosis of the jaw, vitamin D deficiency, wound healing

## Abstract

Osteonecrosis of the jaw (ONJ) is a multifactorial disorder characterized by impaired bone remodeling, vascular compromise, immune dysregulation, and mucosal barrier disruption. Although these mechanisms have been extensively investigated, they are often discussed separately, limiting an integrated understanding of ONJ pathogenesis. Vitamin D has emerged as a biologically relevant factor across these interconnected pathways, yet its role in ONJ remains incompletely defined. This narrative and hypothesis-generating review synthesizes current mechanistic, preclinical, observational, and clinical evidence regarding vitamin D biology and ONJ and proposes a vitamin D-centered vulnerability model in which vitamin D deficiency acts as a context-dependent modifier rather than a primary causal driver. Mechanistically, vitamin D deficiency may impair osteoblast function and mineralization, disrupt angiogenic responses, promote pro-inflammatory immune signaling, and compromise mucosal integrity, collectively creating a microenvironment susceptible to impaired healing and osteonecrosis. These effects are likely to vary across clinical settings, particularly in patients receiving antiresorptive or antiangiogenic therapies. Clinical and epidemiological studies have reported associations between low vitamin D status and increased ONJ risk or severity, while some observational studies suggest that vitamin D supplementation may be associated with improved outcomes in selected populations. However, current human evidence remains predominantly observational and subject to substantial heterogeneity and residual confounding, and direct randomized evidence is lacking. Overall, this framework provides an integrated perspective linking vitamin D biology to ONJ-related pathogenic processes and may support future mechanistic research, risk stratification, and supportive multidisciplinary management strategies. Nevertheless, the proposed model should be interpreted cautiously as hypothesis-generating and requires further validation in well-designed prospective studies and randomized controlled trials.

## 1. Introduction

Osteonecrosis of the jaw (ONJ) is characterized by exposed necrotic bone in the maxillofacial region that fails to heal over a prolonged period, often leading to pain, infection, and functional impairment. The condition comprises a heterogeneous group of disorders with distinct etiologies, most notably osteoradionecrosis (ORN) and medication-related osteonecrosis of the jaw (MRONJ), which present with similar clinical features but arise from different underlying mechanisms [1]. In current clinical practice, MRONJ accounts for the majority of cases and is defined by exposed bone or bone that can be probed through a fistula persisting for more than 8 weeks in patients with a history of exposure to antiresorptive or antiangiogenic agents and no prior radiation therapy to the jaws [2,3]. Originally described in association with bisphosphonates, the definition has since expanded to include other drug classes such as denosumab and antiangiogenic agents, reflecting a broader spectrum of implicated therapies [3].

MRONJ remains an uncommon but clinically significant complication, with substantially higher incidence observed in oncology populations receiving high-dose antiresorptive therapy compared with patients treated for osteoporosis [2,4]. Despite these established clinical associations, the pathogenesis of ONJ remains incompletely understood. Current evidence supports a multifactorial process involving impaired bone remodeling, vascular compromise, immune dysregulation, and disruption of the oral mucosal barrier, which together contribute to impaired tissue repair and susceptibility to necrosis [5,6,7]. Within this framework, systemic factors that modify host susceptibility have gained increasing attention. Vitamin D is of particular interest given its broad roles in skeletal, immune, and epithelial biology, and its deficiency is frequently observed in populations at risk of ONJ [8,9]. In this context, we propose a “vitamin D-centered vulnerability model,” in which vitamin D deficiency functions as a context-dependent, cross-pathway modifier influencing bone, vascular, immune, and mucosal processes rather than serving as a primary causal factor.

The aim of this review is to provide an integrated overview of the role of vitamin D in ONJ. We summarize current understanding of ONJ pathogenesis, examine epidemiological and clinical evidence linking vitamin D status to ONJ risk, and discuss potential mechanisms through which vitamin D may modulate bone remodeling, immune responses, angiogenesis, and mucosal healing in the jaw. We also address the clinical implications of vitamin D assessment and supplementation in the prevention and management of ONJ and highlight key areas for future research.

To achieve these aims, the following search strategy was employed. This article was designed as a narrative review intended to provide an integrated and mechanism-oriented overview of the potential role of vitamin D in ONJ. Relevant literature was identified through selective searches of PubMed/MEDLINE and Scopus using combinations of keywords related to ONJ, MRONJ, osteoradionecrosis, vitamin D, bone remodeling, angiogenesis, immune regulation, and mucosal healing. The review primarily considered English-language studies published up to January 2026, with emphasis on clinical, epidemiological, translational, and mechanistic evidence relevant to ONJ pathogenesis and vitamin D biology. Given the multifactorial and hypothesis-generating nature of the topic, a narrative review approach was considered appropriate for integrating evidence across diverse biological domains.

## 2. Vitamin D Biology: Relevance to Bone Homeostasis and ONJ Susceptibility

This section summarizes key aspects of vitamin D physiology relevant to bone remodeling, mineral homeostasis, and jaw-specific susceptibility to ONJ/MRONJ, to provide biological context for the proposed framework.

### 2.1. Vitamin D Metabolism and Activation

Vitamin D requires sequential hydroxylation in the liver and kidney to generate the biologically active form, 1,25-dihydroxyvitamin D [1,25(OH)_2_D] [10,11].

This activation process is tightly regulated by parathyroid hormone, fibroblast growth factor 23 (FGF23), and mineral homeostasis [12,13]. In addition to renal activation, extra-renal expression of activating enzymes has been identified in osteoblasts, immune cells, and epithelial tissues, enabling tissue-specific production of active vitamin D and local regulation of its biological effects [10,14]. These tissue-specific activation pathways may be particularly relevant in ONJ, where coordinated interactions among bone, immune, and mucosal compartments are critical for tissue repair and healing.

### 2.2. Distribution and Functional Roles of the Vitamin D Receptor

The biological effects of calcitriol are mediated through the vitamin D receptor (VDR), a nuclear receptor expressed across multiple tissues [15,16]. In bone, VDR signaling regulates osteoblast function, bone formation, and mineralization, while effects on osteoclast activity are mediated indirectly through osteoblast-derived signals [17]. VDR is also expressed in immune cells, including macrophages, T lymphocytes, and dendritic cells, where it modulates both innate and adaptive immune responses [18,19]. In the oral environment, VDR expression in keratinocytes and mucosal tissues contributes to epithelial barrier integrity and wound healing [20,21]. This broad distribution across skeletal, immune, and mucosal systems supports the concept that altered vitamin D signaling may influence multiple biological pathways implicated in ONJ pathogenesis.

### 2.3. Genomic and Non-Genomic Actions of Vitamin D

Vitamin D exerts its biological effects through both genomic and non-genomic signaling pathways. In the genomic pathway, calcitriol binds to nuclear VDR and regulates transcription of genes involved in mineral metabolism, bone remodeling, and immune regulation [22,23]. Non-genomic signaling involves rapid intracellular responses associated with calcium flux, kinase activation, cell migration, and early adaptive responses [24,25]. Through these coordinated signaling pathways, vitamin D may influence biological processes relevant to ONJ, including bone remodeling, inflammatory regulation, angiogenesis, and mucosal repair.

### 2.4. Calcium–Phosphate Homeostasis and Bone Mineralization

Vitamin D plays an important role in maintaining calcium and phosphate balance and supporting skeletal mineralization [26]. Through VDR signaling in osteoblasts, vitamin D regulates matrix protein expression and modulates the receptor activator of nuclear factor kappa-B ligand (RANKL)/osteoprotegerin (OPG) axis, thereby influencing the coupling of bone formation and resorption [17,27]. Bone mineralization is also influenced by other micronutrients, particularly vitamin K, which contributes to osteocalcin activation and extracellular matrix mineralization [28,29].

In states of deficiency, reduced calcium availability leads to secondary hyperparathyroidism, increased bone resorption, and impaired mineralization [30]. These changes can result in structurally weakened bone and reduced adaptive remodeling capacity. In the jaw, where physiological remodeling demand is high, impaired mineralization and reduced skeletal adaptability may increase susceptibility to delayed healing and localized osteonecrosis.

### 2.5. Jaw-Specific Physiology and Susceptibility to Injury

The jaw exhibits several anatomical and physiological characteristics that distinguish it from other skeletal sites and contribute to its susceptibility to osteonecrosis [5,7]. Alveolar bone undergoes continuous remodeling in response to mechanical loading, tooth eruption, and periodontal stress, requiring tightly coordinated osteoblast–osteoclast coupling [31,32,33].

In addition, the vascular supply of the jaw is relatively constrained and displays features of limited collateralization, particularly in the mandible, which contains dense cortical bone with comparatively reduced perfusion [32,34]. The jaw is also in constant proximity to the oral microbiological environment, making mucosal barrier integrity essential for preventing bacterial access to the underlying bone [35,36]. Alterations in the oral microbiome and local inflammatory responses have also been associated with ONJ progression [37].

The combination of high remodeling demand, limited vascular reserve, and continuous microbial exposure creates a uniquely vulnerable microenvironment. Within this context, vitamin D deficiency may act as a biologically relevant modifying factor, potentially impairing bone remodeling, immune defense, and mucosal healing, although direct causal relationships with ONJ development remain unproven.

## 3. Pathogenesis of ONJ: A Mechanistic Framework

This section outlines the core pathogenic framework of ONJ, delineating the major interacting processes—impaired bone remodeling, vascular compromise, immune dysregulation, and mucosal barrier disruption—considered independently of vitamin D status. Together, these pathways establish the biological basis through which systemic and local factors may influence ONJ susceptibility and progression. Within this multifactorial context, vitamin D deficiency is discussed as a potentially relevant biological modifier rather than a proven primary cause of ONJ.

### 3.1. Impaired Bone Remodeling and Turnover

Bone integrity depends on tightly coupled osteoclast-mediated resorption and osteoblast-driven formation. In ONJ, this balance is disrupted [38,39]. Antiresorptive agents, particularly bisphosphonates and denosumab, reduce osteoclast activity and slow bone turnover by inhibiting osteoclast differentiation, function, and survival [40]. While therapeutically beneficial in conditions of excessive bone loss, prolonged suppression limits the removal of damaged bone and allows microstructural defects to accumulate, resulting in a low-turnover state with reduced capacity for repair [2,41].

Bone formation may also be compromised. Experimental and observational evidence suggests that vitamin D deficiency may impair osteoblast differentiation, matrix production, and mineralization, potentially reducing regenerative capacity within an already suppressed remodeling environment [8,42]. In preclinical models, vitamin D deficiency has been associated with increased susceptibility to ONJ-like lesions following antiresorptive exposure and local injury [43].

The resulting bone microenvironment is characterized by reduced turnover, impaired remodeling, and diminished adaptive capacity, particularly in the jaw, where physiological remodeling demand is high.

### 3.2. Vascular Insufficiency and Angiogenesis Suppression

Bone viability depends on an adequate microvascular supply. In ONJ, several mechanisms may compromise perfusion. Antiresorptive agents have been associated with impaired endothelial function, reduced angiogenic signaling, and alterations in local microvascular architecture [5,34,44]. Anti-angiogenic therapies, particularly those targeting vascular endothelial growth factor (VEGF) signaling, further inhibit neovascularization and have been independently associated with ONJ [45,46,47]. Combined exposure may intensify these effects and is linked to more severe disease [34].

Structural microvascular changes, including reduced vessel density, disorganized networks, and tissue hypoxia, have been observed in experimental and clinical settings [44,48]. These effects are particularly relevant in the jaw, where vascular reserve is limited and collateral circulation is less developed [49,50].

Vitamin D has also been implicated in endothelial function and vascular homeostasis. Mechanistic studies suggest that vitamin D deficiency may impair angiogenic responses and endothelial signaling; however, direct evidence linking these effects to ONJ development in humans remains limited.

### 3.3. Immune Dysregulation and Impaired Innate Immunity

The oral cavity is continuously exposed to microbial challenges, requiring tightly regulated local immune responses. In ONJ, this balance appears to be disrupted, with evidence of impaired innate immunity and persistent inflammatory activation [38,51,52]. Macrophage responses may remain skewed toward a pro-inflammatory phenotype, with limited transition toward resolution. Altered macrophage polarization and reduced phagocytic function contribute to sustained inflammation and incomplete microbial clearance [40,53,54].

Vitamin D plays a role in innate immune regulation, including the induction of antimicrobial peptides such as cathelicidin antimicrobial peptide (LL-37) and modulation of inflammatory signaling pathways [55,56]. Reduced activity in this pathway may weaken host defense and permit bacterial persistence in exposed tissues. At the same time, dysregulated cytokine signaling may maintain a chronic inflammatory environment that impairs healing [40,51].

Current evidence supporting these interactions is derived primarily from mechanistic and translational studies rather than direct clinical intervention data in ONJ populations.

### 3.4. Oral Mucosal Barrier Dysfunction

The oral mucosa provides a critical barrier protecting underlying bone from the external environment. Disruption of this barrier is often an early event in ONJ, particularly following dental procedures or local tissue injury [51,57,58]. Impaired epithelial repair may expose bone surfaces to microbial colonization and sustain local inflammation.

Antiresorptive agents have been shown to affect oral soft tissues directly, including reduced keratinocyte and fibroblast proliferation, impaired migration, and increased apoptosis, contributing to delayed re-epithelialization [59,60,61]. These effects may be further enhanced by anti-angiogenic therapies, which impair cellular migration and tissue repair [62,63].

Vitamin D contributes to epithelial integrity and keratinocyte function. Deficiency may delay mucosal healing and increase the likelihood of persistent defects [64,65]. Experimental data suggest that active vitamin D analogues may improve soft tissue healing and reduce local inflammation following dental injury [66].

However, direct clinical evidence demonstrating that vitamin D supplementation independently prevents ONJ-related mucosal complications remains insufficient.

### 3.5. Microbial Dysbiosis and Oral Microbiome Perturbations

Once bone becomes exposed, microbial colonization is almost inevitable. Necrotic bone provides a surface that supports dense biofilm formation, which is resistant to clearance and contributes to persistent infection [33,67]. In ONJ, microbial communities enriched with anaerobic species such as *Actinomyces* and *Fusobacterium* have been associated with sustained inflammation and impaired bone repair.

Emerging evidence suggests that microbial imbalance may precede lesion development rather than represent a purely secondary phenomenon. Shifts in microbial community structure, including expansion of pathogenic species and loss of protective flora, have been implicated in disease initiation [68,69,70]. Longitudinal data indicate that these changes may occur prior to overt tissue breakdown [71].

Vitamin D–dependent immune pathways may influence this process. Impaired antimicrobial peptide activity and altered immune regulation may facilitate microbial dysbiosis and sustain a pro-inflammatory microenvironment [69,72]. At present, however, these relationships remain largely mechanistic and associative rather than causally established in human ONJ.

### 3.6. Genetic Susceptibility and VDR Polymorphisms

Not all individuals exposed to similar risk factors develop ONJ, highlighting an important role for host susceptibility. Genetic studies support a polygenic model involving pathways related to bone remodeling, immune regulation, inflammation, and angiogenesis [5,73,74,75].

Among these, polymorphisms in the VDR are of particular interest. Variants affecting VDR expression or function have been associated with differences in bone density, fracture risk, and susceptibility to osteonecrosis [76,77,78]. These alterations may influence downstream signaling across bone, immune, and epithelial systems.

Importantly, circulating vitamin D levels may not fully reflect biological activity, as receptor-level variation can modify cellular responsiveness [77,79]. Although evidence specific to ONJ remains limited and somewhat inconsistent, these findings suggest that inter-individual variation in vitamin D responsiveness may contribute to ONJ susceptibility within a multifactorial framework.

**Figure 1:**
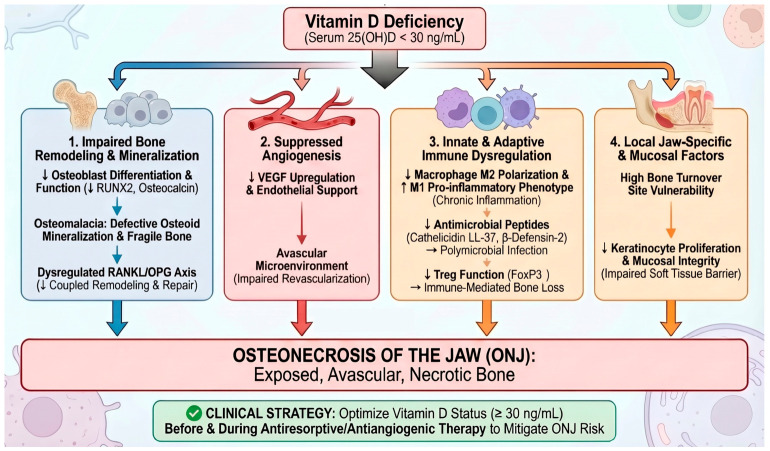
Proposed role of vitamin D deficiency in the pathogenesis of osteonecrosis of the jaw (ONJ). Vitamin D deficiency may contribute to ONJ susceptibility through multiple interconnected biological pathways, including impaired bone remodeling and mineralization, suppression of angiogenesis, immune dysregulation, and disruption of local jaw-specific mucosal integrity. In bone tissue, reduced osteoblast differentiation and mineralization-associated signaling, including RUNX2- and osteocalcin-related pathways, may contribute to defective osteoid mineralization and impaired skeletal repair. Concurrent suppression of vascular support, dysregulated inflammatory responses, reduced antimicrobial defense, and compromised mucosal healing may collectively create a microenvironment susceptible to exposed, avascular, and necrotic bone, particularly in patients receiving antiresorptive or antiangiogenic therapies. Abbreviations: FoxP3, forkhead box P3; LL-37, cathelicidin antimicrobial peptide LL-37; ONJ, osteonecrosis of the jaw; OPG, osteoprotegerin; RANKL, receptor activator of nuclear factor kappa-B ligand; RUNX2, runt-related transcription factor 2; Treg, regulatory T cell; VEGF, vascular endothelial growth factor. ↑, increased; ↓, decreased.

In summary, these interconnected pathways—including bone remodeling, vascular integrity, immune regulation, mucosal barrier function, and host susceptibility—form a multifactorial framework for ONJ pathogenesis, as summarized in Figure 1. Current evidence supporting the role of vitamin D in these pathways is strongest at the mechanistic and preclinical levels, whereas direct clinical evidence remains largely observational and requires further validation.

## 4. Clinical and Epidemiological Evidence Linking Vitamin D Deficiency to ONJ

Epidemiological and clinical observations increasingly suggest an association between vitamin D deficiency and ONJ risk. However, most currently available evidence is observational, and causal relationships have not been definitively established. This section summarizes population-level associations, clinical observations, and important limitations in the existing evidence base.

### 4.1. Epidemiological and Clinical Evidence

Vitamin D deficiency is widely observed in patients with ONJ, with several observational studies reporting elevated rates that often exceed those observed in matched populations exposed to similar antiresorptive therapies without developing the disease. In many cohorts, a substantial proportion of patients present with 25(OH)D levels below 20 ng/mL at diagnosis, with deficiency rates frequently ranging from 70% to 80% [8,80,81]. When broader thresholds are applied, such as 25(OH)D levels below 30 ng/mL, the majority of ONJ patients fall within the insufficient range, indicating that suboptimal vitamin D status is widespread in this population [8,82].

These findings, however, are not uniform across studies. Some case–control studies report lower mean 25(OH)D levels in MRONJ patients compared with controls (approximately 20.5 vs. 29.5 ng/mL) [80], with smaller cohorts describing even lower levels in more severe disease (around 10 ng/mL) [83,84]. In contrast, larger matched analyses have reported similar rates of vitamin D deficiency between groups and did not identify deficiency as an independent risk factor [85]. Consistent with these discrepancies, a systematic review by the European Calcified Tissue Society concluded that the current evidence linking vitamin D deficiency to MRONJ risk remains limited and inconsistent [82].

Across observational cohorts, patients with MRONJ frequently exhibit lower circulating 25(OH)D levels than antiresorptive-treated controls without ONJ [42,80,81,83,86]. In addition, vitamin D deficiency is often unrecognized prior to ONJ onset, as baseline assessment and correction are not routinely performed in clinical practice [8,42].

Overall, current clinical evidence primarily supports an observational association between low vitamin D status and ONJ rather than a definitive causal relationship. Interpretation of these findings should therefore remain cautious given the multifactorial nature of ONJ and the potential influence of confounding factors, including nutritional status, comorbidities, cancer severity, and concurrent therapies. In addition, discrepancies across studies may reflect differences in study design, patient populations, antiresorptive exposure, vitamin D threshold definitions, baseline dental preventive care, and variability in ONJ risk assessment protocols.

### 4.2. Populations at Heightened Risk

Several patient populations exhibit both a high prevalence of vitamin D deficiency and increased baseline susceptibility to ONJ. However, whether vitamin D deficiency independently contributes to ONJ risk in these settings remains uncertain.

#### 4.2.1. Oncology Patients

Oncology patients receiving bone-targeted therapies represent the highest-risk population for ONJ. Vitamin D deficiency is prevalent in this group, with reported prevalence ranging from approximately 30% to over 90%, depending on cancer type and treatment setting [87,88]. Contributing factors include reduced sun exposure, malnutrition, and treatment-related gastrointestinal or organ dysfunction, all of which may limit vitamin D availability. These patients are also frequently exposed to antiresorptive and antiangiogenic agents, placing them in a higher-risk clinical context for ONJ [38,89]. Importantly, current evidence does not clearly distinguish whether vitamin D deficiency independently increases ONJ susceptibility or primarily reflects overall disease burden and systemic frailty in oncology populations.

#### 4.2.2. Elderly Individuals

Vitamin D deficiency is common in older adults, largely due to reduced cutaneous synthesis, limited sunlight exposure, decreased dietary intake, and age-related changes in metabolism. Institutionalized individuals are particularly vulnerable, with consistently high rates reported across multiple cohorts [90,91,92]. These population-level factors often coexist with comorbidities and reduced physiological reserve. Because older individuals are also more likely to receive antiresorptive therapy and undergo invasive dental procedures, disentangling the independent contribution of vitamin D deficiency to ONJ risk remains challenging.

#### 4.2.3. Chronic Kidney Disease

Patients with chronic kidney disease (CKD) frequently exhibit impaired vitamin D metabolism and disturbances in mineral homeostasis associated with CKD–mineral and bone disorder (CKD–MBD) [93,94]. These abnormalities are linked to altered bone turnover and reduced skeletal adaptability. In clinical settings where antiresorptive therapy is used, particularly denosumab, this population is often considered at increased risk of skeletal complications, including ONJ [95,96]. However, CKD represents a complex metabolic and inflammatory condition in which multiple CKD–MBD abnormalities coexist, making it difficult to isolate the specific contribution of vitamin D deficiency to ONJ susceptibility.

#### 4.2.4. Glucocorticoid Use

Long-term glucocorticoid therapy is associated with alterations in calcium balance and reduced bone formation, partly mediated through effects on vitamin D–related pathways [97,98]. Patients receiving chronic glucocorticoids often require antiresorptive treatment for fracture prevention, placing them within a higher-risk clinical context. Vitamin D deficiency is frequently observed in this population and may coexist with other risk factors. Nevertheless, glucocorticoid exposure itself exerts substantial effects on bone remodeling, immune regulation, and wound healing, making causal attribution to vitamin D deficiency alone difficult.

Taken together, these populations illustrate the complex clinical contexts in which vitamin D deficiency coexists with multiple established ONJ risk factors. Current evidence therefore supports vitamin D deficiency primarily as a potentially relevant cofactor or biomarker of vulnerability rather than a proven independent determinant of ONJ development.

The potential biological mechanisms through which vitamin D may influence ONJ pathogenesis are discussed in the following section.

## 5. Vitamin D as a Cross-Pathway Modifier in ONJ

**Figure 2:**
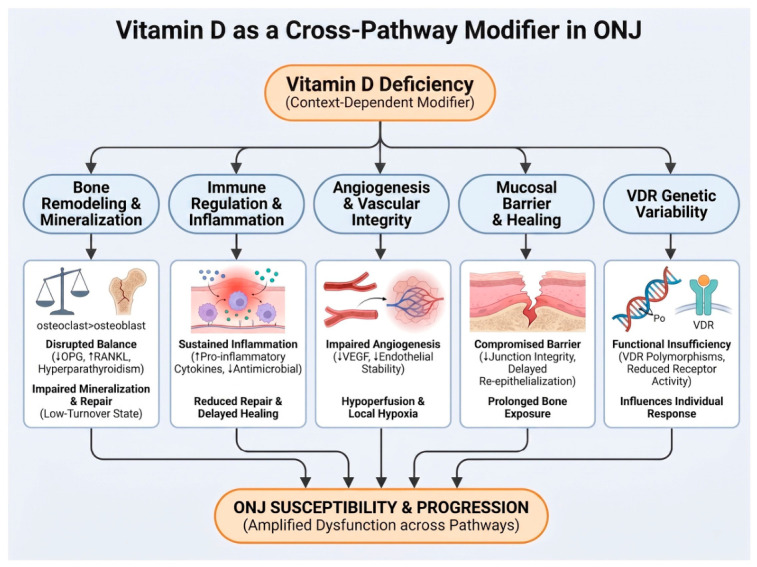
Proposed mechanisms through which vitamin D deficiency may influence ONJ susceptibility and progression. Vitamin D deficiency may affect multiple biological domains relevant to ONJ, including bone remodeling and mineralization, immune regulation and inflammation, angiogenesis and vascular integrity, mucosal barrier healing, and VDR-related functional responsiveness. These interconnected effects may collectively contribute to impaired tissue repair and increased ONJ susceptibility within susceptible clinical contexts. Abbreviations: ONJ, osteonecrosis of the jaw; OPG, osteoprotegerin; RANKL, receptor activator of nuclear factor kappa-B ligand; VEGF, vascular endothelial growth factor; VDR, vitamin D receptor. ↑, increased; ↓, decreased.

This section examines the specific mechanisms through which vitamin D may modulate each of the pathogenic pathways outlined in Section 3, as illustrated in Figure 2. Evidence in this area derives predominantly from mechanistic, translational, and preclinical studies; direct clinical data remain limited.

### 5.1. Bone Remodeling and Mineralization

Bone remodeling in the jaw depends on the balance between osteoclast-mediated resorption and osteoblast-driven formation, a process regulated in part by the receptor activator of nuclear factor kappa-B (RANK)/RANKL/OPG axis. Vitamin D contributes to this system by modulating osteoblast-derived RANKL and OPG expression, thereby supporting coordinated remodeling under physiological conditions [99,100].

When vitamin D status is inadequate, this balance is disrupted. Reduced OPG expression and secondary hyperparathyroidism shift signaling toward increased osteoclast activity, while osteoblast differentiation and matrix mineralization are impaired [101]. The result is uncoupled remodeling and the accumulation of structurally compromised bone.

This imbalance may be further accentuated by antiresorptive therapy, which suppresses osteoclast function and limits turnover, restricting clearance of microdamage [102,103]. Mechanistically, impaired vitamin D signaling may reduce compensatory bone formation within this low-turnover environment; however, direct clinical evidence linking these pathways to ONJ development remains limited.

### 5.2. Immune Regulation and Inflammatory Modulation

Immune regulation is essential for maintaining tissue integrity in the jaw, where continuous microbial exposure requires tightly controlled inflammatory responses. Vitamin D contributes to this balance by influencing macrophage polarization, antimicrobial peptide production, and cytokine signaling. Under physiological conditions, calcitriol promotes a shift toward a reparative macrophage phenotype and enhances antimicrobial defense through induction of peptides such as LL-37 and β-defensins [104,105,106,107].

In vitamin D deficiency, this regulatory system is disrupted. Macrophages may remain in a pro-inflammatory state, sustaining cytokine production and limiting resolution, while reduced antimicrobial peptide expression weakens host defense and permits microbial persistence [56,104]. Vitamin D signaling has also been associated with suppression of pro-inflammatory mediators, including interleukin-1β, tumor necrosis factor alpha (TNF-α), interleukin-6, and interleukin-17, together with support of regulatory T-cell responses [105,108,109].

These observations provide biological plausibility for a role of vitamin D in inflammatory regulation relevant to ONJ; however, supporting evidence is derived primarily from mechanistic and translational studies rather than direct interventional studies in ONJ populations.

### 5.3. Angiogenesis and Vascular Integrity

Adequate vascular supply is essential for bone viability and repair, particularly in the jaw, where perfusion is relatively constrained. Vitamin D supports vascular integrity through regulation of angiogenic signaling and endothelial function. Calcitriol has been shown to modulate VEGF expression and hypoxia-related pathways while also preserving endothelial homeostasis through effects on nitric oxide availability and oxidative stress [110,111,112].

When vitamin D status is inadequate, both angiogenic capacity and endothelial function may be impaired. Reduced VEGF signaling and disrupted hypoxia responses may limit neovascularization, while endothelial dysfunction promotes a hypoperfused microenvironment [110,111].

These effects may be particularly relevant in patients receiving anti-angiogenic therapy, where VEGF inhibition already restricts vascular repair. Nevertheless, current evidence linking vitamin D–related vascular effects directly to ONJ progression in humans remains largely indirect and mechanistic.

### 5.4. Mucosal Barrier Function and Wound Healing

The oral mucosa serves as a critical barrier protecting underlying bone from microbial exposure. Vitamin D contributes to mucosal integrity through its effects on keratinocytes, which express the VDR and are capable of local activation of vitamin D. This signaling supports epithelial differentiation, intercellular junction stability, and antimicrobial defense [113,114]. In addition, vitamin D promotes keratinocyte migration and re-epithelialization during wound healing [115].

In vitamin D deficiency, these processes are compromised. Disruption of epithelial integrity increases permeability, while impaired keratinocyte proliferation and migration delay mucosal closure. These defects are particularly relevant following dental procedures, where effective epithelial healing is required to protect underlying bone.

When combined with antiresorptive therapy, healing may be further delayed, allowing prolonged bone exposure and facilitating microbial colonization [8,42,84]. Although these observations support a biologically plausible role for vitamin D in mucosal healing, direct evidence demonstrating that vitamin D optimization independently prevents ONJ-related tissue breakdown remains insufficient.

### 5.5. VDR Genetic Variability and Functional Responsiveness

Variability in vitamin D responsiveness is influenced not only by circulating 25(OH)D levels but also by genetic variation in the VDR. Common polymorphisms, including FokI, BsmI, TaqI, and ApaI, may alter receptor activity, expression, and downstream signaling efficiency [77,116,117,118].

These variants have been associated with differences in bone mineral density, inflammatory regulation, and response to antiresorptive therapy, suggesting that individuals with similar vitamin D levels may exhibit different biological responses. In the context of ONJ, reduced VDR function may theoretically attenuate vitamin D–mediated regulatory effects across bone, immune, vascular, and mucosal systems.

However, clinical findings regarding VDR polymorphisms and ONJ susceptibility remain limited and inconsistent, and the clinical relevance of these genetic associations has not yet been established.

## 6. Vitamin D Across Clinical ONJ Subtypes

The relevance of vitamin D in ONJ may vary across clinical subtypes and treatment settings. The following sections summarize how vitamin D deficiency may interact with dominant pathological processes in major ONJ subtypes, including impaired bone remodeling, vascular compromise, immune dysregulation, and tissue injury. Current evidence supporting these subtype-specific interactions is derived primarily from mechanistic studies, observational associations, and indirect clinical evidence rather than definitive causal data.

### 6.1. Bisphosphonate-Related Osteonecrosis of the Jaw

In bisphosphonate-related osteonecrosis of the jaw (BP-MRONJ), the dominant clinical context is sustained suppression of bone turnover due to osteoclast inhibition and prolonged drug retention within bone. Under these conditions, the capacity for adaptive remodeling is inherently limited. Vitamin D deficiency does not initiate this process but may amplify its consequences by further impairing osteoblast function and matrix mineralization, thereby creating a mismatch between suppressed resorption and insufficient bone formation and contributing to the accumulation of structurally compromised bone, particularly in the jaw where remodeling demand is high [43,119]. This interaction has been described as a metabolic mismatch, in which antiresorptive therapy amplifies pre-existing defects in bone quality, including features consistent with osteomalacia [8].

In addition, bisphosphonates may directly affect oral keratinocytes and soft tissue repair, contributing to delayed mucosal healing and increased susceptibility to bone exposure following local injury [4]. Clinical observations further support this interaction, with lower 25(OH)D levels frequently reported in BP-MRONJ patients, although the strength of association varies across studies [83,84].

Taken together, available evidence supports a biologically plausible interaction between vitamin D deficiency and BP-MRONJ pathophysiology. In the current state of evidence, vitamin D deficiency is best regarded as a biologically plausible amplifier of BP-MRONJ-related bone quality deficits, pending confirmation in prospective clinical studies.

### 6.2. Denosumab-Related ONJ

Denosumab-related ONJ arises in a distinct clinical context characterized by profound but reversible suppression of bone turnover through inhibition of RANKL signaling [120,121]. Unlike bisphosphonates, its effects are not retained in bone, resulting in a dynamic system in which both suppression during treatment and rebound activation after discontinuation may destabilize skeletal homeostasis [122,123].

Vitamin D status plays a key modulatory role in maintaining metabolic stability. Deficiency reduces intestinal calcium absorption and increases susceptibility to hypocalcemia during active therapy, a well-recognized complication in denosumab-treated patients [124]. This results in a metabolically constrained state in which both mineral balance and adaptive remodeling capacity are compromised. Following discontinuation, rebound osteoclast activation may further stress structurally vulnerable bone and contribute to delayed healing or lesion progression [125].

Clinical practice guidelines therefore recommend correction of vitamin D deficiency and calcium imbalance before and during denosumab therapy. Correction of vitamin D deficiency represents a clinically reasonable supportive measure in denosumab-treated patients, primarily to maintain metabolic stability, though its specific role in ONJ prevention has yet to be demonstrated in controlled trials.

### 6.3. Antiangiogenic Agent-Related ONJ

Antiangiogenic agent–related ONJ develops in a vascular-dominant context in which inhibition of VEGF signaling impairs neovascularization and tissue perfusion. Anti-VEGF monoclonal antibodies, tyrosine kinase inhibitors, and mechanistic target of rapamycin (mTOR) inhibitors disrupt endothelial proliferation and microvascular integrity, resulting in a hypoxic and hypoperfused tissue environment [45,126].

In this setting, vitamin D deficiency appears to interact with, rather than duplicate, these vascular effects. By reducing VEGF expression and impairing endothelial nitric oxide signaling, deficiency may further limit vascular adaptability and angiogenic reserve [112,127]. This compounded impairment is particularly relevant in the jaw, where vascular supply is already constrained and tolerance to ischemic stress is reduced [34,41].

Clinically, ONJ risk appears highest when antiangiogenic agents are combined with antiresorptive therapies [128,129]. Whether vitamin D deficiency meaningfully amplifies ONJ risk in the antiangiogenic setting remains an open question that warrants dedicated investigation in this patient population.

### 6.4. Osteoradionecrosis

Osteoradionecrosis represents a distinct pathological context characterized by radiation-induced vascular injury and chronic tissue hypoxia. Progressive endarteritis leads to reduced perfusion, depletion of osteogenic cells, and impaired remodeling capacity, resulting in a hypoxic, hypovascular, and hypocellular environment [130,131,132]. These changes are further compounded by fibrosis and chronic inflammation, which limit tissue repair and regeneration [133,134].

Within this already compromised environment, vitamin D deficiency may further reduce residual regenerative capacity by impairing osteoblast function, weakening immune responses, and limiting endothelial support mechanisms [21,135]. Although direct clinical evidence linking vitamin D to ORN risk remains limited, data from MRONJ studies and experimental models suggest that deficiency acts as a compounding factor that exacerbates impaired healing rather than serving as a primary cause [43,81].

Given the high prevalence of vitamin D deficiency among patients undergoing head and neck radiotherapy, optimizing vitamin D status in this population may be clinically reasonable as part of broader supportive care, even in the absence of direct evidence specifically linking deficiency to ORN development.

## 7. Clinical Evidence: Vitamin D Supplementation and ONJ Outcomes

At the clinical level, the potential role of vitamin D supplementation in modifying ONJ risk and outcomes has been explored in observational, translational, and preclinical studies. However, direct clinical evidence demonstrating that vitamin D supplementation independently prevents ONJ or improves major clinical outcomes remains limited. This section summarizes the current evidence while distinguishing observational associations, preclinical findings, and areas where causal inference remains uncertain.

### 7.1. Observational Studies: Supplementation and ONJ Risk Reduction

Observational studies constitute the largest body of clinical evidence examining the relationship between vitamin D supplementation and ONJ outcomes. Across diverse populations—including oncology patients receiving intravenous bisphosphonates, osteoporosis patients receiving oral antiresorptives, and individuals treated with denosumab—adequate vitamin D supplementation, often combined with calcium, has been associated with lower reported ONJ incidence [42,84]. In retrospective oncology cohorts, patients receiving concurrent supplementation demonstrated lower MRONJ rates than unsupplemented individuals, with some studies reporting a two- to fourfold difference in incidence [86].

Some of these associations remained significant after adjustment for drug exposure, cancer type, and dental risk factors [8]. Cross-sectional and case–control studies further show that patients with ONJ tend to have lower circulating 25(OH)D levels [42], with vitamin D deficiency associated with increased ONJ risk in some cohorts [84,86].

Similar observational patterns have been reported in osteoporosis populations and pharmacovigilance databases, where co-prescription of vitamin D and calcium has been associated with lower MRONJ reporting rates compared with antiresorptive therapy alone [8]. In denosumab-treated patients, adequate vitamin D status has additionally been associated with lower rates of hypocalcemia [42]. Several observational analyses further suggest that patients achieving circulating 25(OH)D levels above approximately 30–32 ng/mL may experience lower ONJ incidence or severity [84].

Prospective observational registries incorporating systematic 25(OH)D monitoring have also reported lower ONJ incidence in settings using protocol-driven vitamin D optimization [82]. However, interpretation of these findings is complicated by concurrent improvements in dental surveillance, preventive care, nutritional support, and overall clinical management.

Importantly, all currently available human data are observational in nature. Residual confounding—including differences in overall health status, nutritional condition, cancer severity, healthcare access, and preventive dental care—may substantially influence these associations. Therefore, current evidence supports a potential association between vitamin D supplementation and reduced ONJ risk but does not establish definitive causality.

### 7.2. Interventional and Preclinical Evidence

In contrast to the relatively extensive observational literature, direct interventional evidence evaluating vitamin D supplementation for ONJ prevention or treatment remains limited. To date, no randomized controlled trials in humans have specifically evaluated vitamin D supplementation as a primary intervention for ONJ prevention or management.

Existing interventional data are largely derived from preclinical models, in which vitamin D status has been experimentally manipulated. In murine models of MRONJ, administration of active vitamin D analogues (e.g., 1,25(OH)_2_D or eldecalcitol) has been shown to reduce the development of osteonecrosis following tooth extraction despite continued exposure to zoledronic acid [66]. Similarly, in rat models, systemic vitamin D_3_ supplementation administered around the time of antiresorptive therapy and dental extraction has been associated with reduced histologic and macroscopic features of osteonecrosis, although effects appear to depend on timing and dosing [136]. Conversely, vitamin D deficiency in combination with bisphosphonate exposure has been shown to increase ONJ prevalence in animal models, with reported rates approaching 60–70% compared with minimal incidence in vitamin D–replete controls [43].

Additional mechanistic studies further support a biologically plausible modulatory role for vitamin D. In a randomized placebo-controlled study in postmenopausal women receiving zoledronic acid, vitamin D status influenced early VEGF-related responses [137]. Experimental studies involving photobiomodulation-induced increases in vitamin D levels or vitamin D–conditioned immune cells have also demonstrated enhanced bone healing and reduced inflammation in jaw injury models [138,139].

Collectively, these findings provide mechanistic and preclinical support for a potential role of vitamin D sufficiency in modifying ONJ-related biological processes. However, in the absence of direct randomized human evidence, vitamin D supplementation should currently be regarded as a supportive and biologically plausible adjunctive strategy rather than a proven ONJ-preventive or therapeutic intervention.

To address these evidence gaps, future studies should evaluate the role of vitamin D in ONJ through well-designed prospective cohorts and randomized controlled trials. In particular, studies conducted in high-risk populations receiving antiresorptive or antiangiogenic therapies may help clarify whether optimization of vitamin D status influences ONJ incidence, severity, or healing outcomes.

### 7.3. Vitamin D Thresholds and Clinical Targets

Serum 25(OH)D levels demonstrate a graded association with ONJ risk, with progressively lower risk observed at higher concentrations rather than a strict threshold effect. Levels < 20 ng/mL have been reported in some studies to be associated with higher ONJ risk and poorer bone quality [80,140], while concentrations between 20 and 30 ng/mL are generally considered insufficient. In contrast, levels ≥ 30 ng/mL are associated with lower MRONJ severity and more favorable bone and oral outcomes [81,141].

Emerging observational evidence has also suggested that levels > 40 ng/mL may be associated with improved healing or lower ONJ severity in selected high-risk populations, although supporting evidence remains limited [81]. Severe deficiency (<10–12 ng/mL) has been associated with the most pronounced skeletal and metabolic abnormalities [142,143].

Because these data are derived primarily from observational studies rather than randomized trials, optimal vitamin D targets specifically for ONJ prevention remain uncertain. Nevertheless, maintaining adequate vitamin D status—commonly ≥30 ng/mL—is generally considered biologically reasonable and clinically appropriate in patients receiving antiresorptive or antiangiogenic therapies, particularly for overall skeletal and metabolic health.

### 7.4. Impact of Vitamin D Status on Disease Severity and Healing

Available evidence suggests that vitamin D status may influence both the severity of disease at presentation and subsequent clinical course in ONJ. Observational studies consistently report lower 25(OH)D levels in patients with MRONJ compared with antiresorptive-treated controls, and deficiency has been associated with more advanced disease stages at diagnosis [20,86]. Higher vitamin D levels (≥30 ng/mL) have been linked to less severe MRONJ at presentation, suggesting a potential role in modulating disease extent rather than initiation alone [81].

The impact of vitamin D on healing outcomes appears less consistent. Some studies suggest that supplementation or higher vitamin D status is associated with improved mucosal healing and reduced severity, particularly when optimized before dental procedures [81]. However, in established MRONJ, supplementation alone has not consistently improved surgical outcomes, and healing may be more strongly influenced by factors such as bone turnover, infection control, and overall nutritional status [144,145].

Supporting evidence from periodontal and experimental models further suggests that vitamin D deficiency is associated with impaired wound healing and increased inflammation [21,146]. Taken together, current evidence supports a potential modulatory role of vitamin D in ONJ severity and tissue healing; however, available data remain largely associative and insufficient to establish vitamin D supplementation as an evidence-based therapeutic strategy for established ONJ.

## 8. Clinical Implications and Management Considerations

From a clinical perspective, vitamin D status represents a potentially modifiable factor that may influence skeletal health, metabolic stability, and tissue healing in patients at risk of ONJ. However, current evidence supporting vitamin D–directed interventions specifically for ONJ prevention or treatment remains limited and largely observational. This section discusses potential clinical implications while emphasizing the supportive and adjunctive nature of current recommendations.

### 8.1. Screening and Risk Assessment

Pre-treatment assessment of vitamin D status represents an important yet underutilized component of ONJ prevention among patients receiving antiresorptive or antiangiogenic therapy. This reflects the high burden of vitamin D deficiency in high-risk populations—including oncology patients, older adults, individuals with CKD, and those receiving long-term glucocorticoids—where deficiency frequently remains unrecognized without laboratory evaluation [80,81].

The window for effective correction is widest prior to therapy initiation. Available evidence suggests that prophylactic vitamin D supplementation before antiresorptive therapy or invasive dental procedures may reduce MRONJ severity, whereas correction after disease onset appears less effective [66,81,147]. This consideration is particularly relevant in settings such as denosumab initiation, where underlying deficiency increases the risk of hypocalcemia and may complicate early management.

Despite these considerations, real-world data indicate that baseline 25(OH)D measurement is performed in fewer than half of eligible patients, highlighting a persistent gap between guideline recommendations and clinical practice [148,149]. Importantly, however, evidence supporting routine vitamin D screening specifically for ONJ prevention remains indirect, and current recommendations are based primarily on biological plausibility, overall skeletal health considerations, and observational associations rather than definitive ONJ-specific clinical trial data.

Overall, early identification and correction of vitamin D deficiency may be considered a supportive component of multidisciplinary risk reduction, particularly in high-risk populations, rather than a proven stand-alone ONJ-preventive strategy.

### 8.2. Supplementation Strategies

Vitamin D supplementation in patients at risk of ONJ should be individualized according to baseline 25(OH)D levels, clinical risk profile, and treatment context. Among available formulations, cholecalciferol (vitamin D_3_) is generally preferred over ergocalciferol (vitamin D_2_), as it produces a greater and more sustained increase in serum 25(OH)D levels across both general and CKD populations [150,151,152]. Ergocalciferol remains an acceptable alternative, although dose adjustment may be required to achieve comparable biochemical responses.

Dosing strategies should be guided by baseline vitamin D status and therapeutic goals. In deficient individuals, higher doses are typically required, often including a loading phase followed by maintenance therapy. Clinical observations suggest that proactive supplementation—particularly in the peri-procedural setting—may reduce MRONJ risk or severity, whereas its impact appears more limited once disease is established [42,81]. However, these findings remain observational and have not been confirmed in randomized clinical trials.

In patients with CKD, nutritional vitamin D alone may be insufficient because impaired renal activation and secondary hyperparathyroidism may limit biological activity [153,154,155,156]. Experimental evidence suggests that active vitamin D analogues may attenuate ONJ-related inflammatory and skeletal changes in preclinical models [66]. Nevertheless, evidence supporting the use of active vitamin D analogues specifically for ONJ prevention or treatment in humans remains limited.

Vitamin D supplementation should also be considered alongside adequate calcium intake to maintain mineral balance and skeletal stability. Vitamin D levels may decline after supplementation is discontinued, particularly in patients with chronic illness, supporting the rationale for periodic monitoring [150,157,158].

Taken together, current evidence supports vitamin D supplementation primarily as a supportive strategy aimed at maintaining metabolic and skeletal stability rather than as an evidence-based intervention specifically proven to prevent ONJ.

### 8.3. Integration into Multidisciplinary Prevention Protocols

Effective ONJ prevention requires a structured, multidisciplinary care pathway rather than reliance on a single intervention. Current guidelines consistently emphasize coordinated management among prescribing physicians, dentists, and oral and maxillofacial surgeons to align antiresorptive therapy, dental care, and long-term surveillance [159,160,161,162,163]. Within this framework, vitamin D optimization should be incorporated as an adjunctive measure alongside dental risk assessment and systemic management.

Pre-treatment dental evaluation remains the most critical component of ONJ prevention. Completion of necessary extractions, periodontal therapy, and stabilization of oral disease prior to antiresorptive therapy initiation significantly reduces MRONJ risk, with reported reductions ranging from approximately 33% to over 70% in preventive cohorts [36,38,160,164]. Adequate healing time before drug initiation is essential to allow mucosal closure and minimize subsequent bone exposure [36,38,165]. Optimization of vitamin D status may further support mucosal healing and enhance the effectiveness of pre-treatment dental interventions.

Long-term prevention depends on sustained oral hygiene and regular dental surveillance. Maintenance protocols focusing on biofilm control, avoidance of mucosal trauma, and early management of dental pathology are consistently recommended to reduce ONJ risk [4,162,166,167,168]. Conservative dental approaches, favoring endodontic treatment over extraction whenever feasible, are central to minimizing procedural risk during therapy.

The role of drug holidays remains uncertain and should be individualized. Current evidence is inconsistent, and decisions must balance the potential benefit in surgical healing against the risk of fracture or cancer-related complications [38,41,161,169]. Similarly, although maintaining adequate vitamin D status is biologically reasonable and may support tissue healing and skeletal health, vitamin D optimization should currently be viewed as an adjunctive component of multidisciplinary prevention rather than an established ONJ-prevention strategy supported by definitive clinical evidence.

### 8.4. Role in the Management of Established ONJ

Management of established ONJ is fundamentally stage-guided and multidisciplinary, with conservative measures and infection control in early stages and surgical intervention in advanced disease. Within this framework, vitamin D should be regarded as an adjunctive component of supportive care rather than a primary therapeutic modality, supporting biological processes relevant to healing without replacing standard care.

Mechanistically, adequate vitamin D status is associated with improved bone metabolism, immune regulation, and mucosal repair. However, current clinical evidence indicates that although vitamin D deficiency is common in MRONJ and correction may support overall tissue response, supplementation alone does not significantly improve healing outcomes once disease is established [42,81]. Its clinical impact therefore appears more pronounced in preventive settings than in the context of active disease.

Therapeutic outcomes in established MRONJ are primarily determined by appropriate surgical management and infection control. Surgical approaches—ranging from sequestrectomy to resection—have been associated with higher rates of mucosal healing and disease resolution, particularly when combined with antibiotics and structured follow-up [170,171,172]. Adjunctive therapies, including teriparatide, hyperbaric oxygen therapy, and photobiomodulation, may provide additional benefit in selected cases, although the evidence remains heterogeneous and limited [173,174,175,176,177].

Within this multimodal framework, maintaining adequate vitamin D status is clinically reasonable and biologically plausible. Nevertheless, current evidence does not demonstrate a clear independent effect of vitamin D supplementation on major clinical endpoints in established ONJ. Accordingly, vitamin D optimization should currently be interpreted as part of comprehensive supportive care rather than a proven therapeutic intervention for established disease.

## 9. Knowledge Gaps and Future Research Directions

Current evidence linking vitamin D status to ONJ is largely observational, and direct clinical evidence remains limited. As such, the overall strength of evidence supporting vitamin D–related interventions in ONJ should be interpreted with caution. Key knowledge gaps span several domains, including causal inference, optimal vitamin D thresholds, inter-individual variability, and translational relevance.

Future research should prioritize prospective studies that integrate clinical, biochemical, dental, and treatment-related variables using standardized assessment protocols. In particular, well-designed longitudinal cohort studies and randomized controlled trials in patients receiving antiresorptive or antiangiogenic therapies will be important to clarify causality and evaluate whether vitamin D optimization influences ONJ incidence, severity, or healing outcomes. Standardized peri-procedural supplementation studies and biomarker-guided risk stratification approaches may also help identify patients most likely to benefit from intervention. In addition, stratified analyses according to treatment type, baseline vitamin D status, underlying disease, and clinical risk profile will likely be necessary to better interpret heterogeneous patient responses.

Another unresolved issue is the appropriate target level of serum 25(OH)D. Existing thresholds are largely based on general skeletal outcomes and may not fully reflect the biological requirements of the jaw, where bone turnover, mucosal integrity, vascular supply, and microbial exposure differ from other skeletal sites. Studies linking circulating vitamin D levels with jaw-specific outcomes, including mucosal healing, infection control, and local bone turnover, may therefore provide more clinically relevant targets.

Inter-individual variation in response to vitamin D is also likely to be important. Polymorphisms in the VDR and related signaling pathways may influence both susceptibility to ONJ and response to supplementation. However, current data are limited by small sample sizes and inconsistent findings. Larger multicenter studies across diverse populations, together with functional tissue-based analyses, will be required before these observations can be translated into clinical application.

Experimental models have provided important mechanistic insights, but their relevance to human disease remains uncertain. Differences in vitamin D metabolism, drug exposure, and oral environment limit direct extrapolation to human disease. Development of experimental models that more closely replicate human ONJ, together with translational studies integrating mechanistic findings with clinical phenotypes, may improve translational relevance.

Emerging evidence also suggests that interactions between vitamin D signaling and the oral microbiome may play a role in ONJ susceptibility. In addition, vitamin D analogues and tissue-selective VDR agonists may allow more targeted modulation of vitamin D pathways while minimizing systemic effects such as hypercalcemia. Further investigation in these areas may help clarify the sources of inter-individual variability in ONJ risk and inform the development of biologically plausible preventive strategies.

## 10. Conclusions

Vitamin D deficiency is common in populations at risk of ONJ and is associated with biological conditions that may influence bone remodeling, vascular integrity, immune regulation, and mucosal healing. Within this framework, vitamin D is best interpreted as a context-dependent modifying factor rather than a primary causal driver of disease. The proposed vitamin D-centered vulnerability model provides an integrated perspective linking vitamin D biology to the multifactorial pathogenesis of ONJ.

From a clinical perspective, assessment and optimization of vitamin D status may represent a reasonable supportive component of multidisciplinary risk management, particularly in high-risk populations. However, current evidence remains predominantly mechanistic and observational, and definitive preventive or therapeutic benefit has not been established. Accordingly, this framework should be regarded as hypothesis-generating and requires validation through well-designed prospective studies and randomized controlled trials. Until stronger evidence becomes available, vitamin D optimization should be considered a biologically plausible adjunctive strategy rather than an established intervention for ONJ prevention or treatment.

## Data Availability

Not applicable.

## Abbreviations

The following abbreviations are used in this manuscript:

1,25(OH)_2_D	1,25-dihydroxyvitamin D
25(OH)D	25-hydroxyvitamin D
BP-MRONJ	Bisphosphonate-related osteonecrosis of the jaw
CKD	Chronic kidney disease
FGF23	Fibroblast growth factor 23
LL-37	Cathelicidin antimicrobial peptide LL-37
MRONJ	Medication-related osteonecrosis of the jaw
mTOR	Mechanistic target of rapamycin
ONJ	Osteonecrosis of the jaw
OPG	Osteoprotegerin
ORN	Osteoradionecrosis
RANK	Receptor activator of nuclear factor kappa-B
RANKL	Receptor activator of nuclear factor kappa-B ligand
RUNX2	Runt-related transcription factor 2
TNF-α	Tumor necrosis factor alpha
VDR	Vitamin D receptor
VEGF	Vascular endothelial growth factor
